# Temporal dynamics of cardiac biomarkers in therapy‑related cardiotoxicity: a prospective cardio‑oncology cohort study

**DOI:** 10.1186/s40959-026-00538-3

**Published:** 2026-07-06

**Authors:** Lidia Anca Kajanto, Dana Lucia Stănculeanu, Anca Chisoi, Nicolae Dobrin, Anita-Cristina Ionescu, Ioan Sîrbu, Ion Alexandru Popovici, Pundiche Mihaela, Ionut Bulbuc, Andreea Diana Kajanto, Bogdan Cîmpineanu, Adrian Neluțu Mitroi, Georgeta-Camelia Cozaru

**Affiliations:** 1 Oncological Institute “Prof. Dr. Alexandru Trestioreanu”, Bucharest, 022328 Romania; 2 Department of Oncology, “Carol Davila” University of Medicine and Pharmacy, Bucharest, 020021 Romania; 3 “Sf. Apostol Andrei” Emergency County Hospital, Tomis Bulevard 145, Constanta, 900591 Romania; 4 Center for Research and Development of the Morphological and Genetic Studies of Malignant Pathology (CEDMOG), “Ovidius” University of Constanta, Constanta, 900591 Romania; 5 Faculty of Dentistry, Carol Davila University of Medicine and Pharmacy, Bucharest, Romania; 6 Faculty of Medicine, “Ovidius” University of Constanta, 1 Universitatii Street, Constanta, 900470 Romania; 7 Targu Mures Clinical Emergency County Hospital, Gheorghe Marinescu 50, Targu Mures, 540136 Romania; 8 Academy of Romanian Scientists, 3 Ilfov Street, Bucharest, 050094 Romania

**Keywords:** Cardio-oncology, Cardiotoxicity, High-sensitivity cardiac troponin T, NT-proBNP, sST2, Chemotherapy-related cardiac dysfunction

## Abstract

**Background:**

Cardiotoxicity associated with antineoplastic therapies remains an important clinical challenge in modern oncology. Early identification of subclinical cardiac injury may improve cardiovascular surveillance and long-term outcomes in cancer patients.

**Methods:**

This prospective observational cohort study included 90 adult patients with breast cancer, lymphoma, or lung cancer receiving potentially cardiotoxic therapy. Patients with clinically manifest heart failure, severe arrhythmias, advanced renal disease, recent acute myocardial infarction, untreated severe valvular disease, or other major cardiovascular comorbidities were excluded. High-sensitivity cardiac troponin T (hs-cTnT), NT-proBNP, and soluble ST2 (sST2) were measured at baseline, during treatment, at therapy completion, and at late follow-up. Non-parametric tests were used for repeated-measures and subgroup comparisons.

**Results:**

Significant temporal variations were observed for all biomarkers. hs-cTnT increased from 5 [3–8] ng/L at baseline to 15 [10–25] ng/L at T2, while NT-proBNP increased from 120 [80–190] pg/mL to 210 [150–320] pg/mL. sST2 demonstrated a delayed elevation and remained increased at follow-up. Distinct biomarker patterns were observed across malignancy subgroups, with higher hs-cTnT increases in breast cancer, more prominent NT-proBNP elevation in lymphoma, and higher sST2 values in lung cancer.

**Conclusions:**

Serial biomarker assessment may provide complementary information on myocardial injury, ventricular stress, and fibrotic remodeling during cancer therapy. The observed biomarker trajectories are suggestive of different biological processes involved in therapy-related cardiotoxicity, but they should be interpreted in conjunction with imaging findings and clinical outcomes.

## Introduction

Cardiotoxicity induced by antineoplastic therapies has become one of the most important challenges in modern oncologic medicine, particularly as cancer survival has improved and cardiovascular complications increasingly influence long-term prognosis [1–3]. With the advent of targeted therapies and immunotherapy, the toxicity profile has diversified beyond classical anthracycline-related injury, requiring closer cardio-oncology monitoring [4–7].

Cardiac injury related to cancer therapy is not a single phenomenon, but rather a pathophysiological continuum involving myocardial injury, hemodynamic stress, inflammation, and fibrotic remodeling [7–9]. Anthracyclines are mainly associated with dose-dependent myocardial injury, anti-HER2 therapies with potentially reversible functional impairment, whereas tyrosine kinase inhibitors and immune checkpoint inhibitors may induce vascular, metabolic, or immune-mediated cardiac toxicity [9–13].

Chemotherapy-related cardiac dysfunction remains clinically relevant, with reported prevalence varying according to treatment type, patient risk profile, and monitoring strategy [7, 14]. Because structural or functional cardiac changes may occur before overt heart failure, early detection of subclinical cardiotoxicity is essential [10, 14, 15].

Molecular biomarkers provide an early, non-invasive, and sensitive warning system that complements imaging investigations. Troponin, NT-proBNP, and sST2 reflect different but complementary biological processes: myocardial injury, ventricular wall stress, and myocardial remodeling/inflammation, respectively [16–19]. However, the temporal behavior of these biomarkers during and after oncologic treatment, and their variation according to malignancy type, remain insufficiently characterized [16, 19].

The aim of this research was to evaluate the temporal dynamics of cardiac biomarkers in patients receiving potentially cardiotoxic antineoplastic therapy. We specifically assessed serial changes in high-sensitivity cardiac troponin T, NT-proBNP, and sST2 at predefined treatment time points, with the objective of identifying distinct biomarker patterns that may support earlier detection of subclinical therapy-related cardiotoxicity.

## Materials and methods

### Study population

This study was designed as a prospective, observational, single-center cohort study conducted at the “Prof. Dr. Alexandru Trestioreanu” Oncology Institute in Bucharest. Patients were consecutively enrolled between 2023 and 2025.

The study included adult patients diagnosed with breast cancer, lymphomas, and lung cancer —malignancies commonly treated with therapeutic regimens associated with a documented risk of cardiotoxicity and grouped into three predefined oncologic subgroups: breast cancer, lymphoma, and lung cancer.

All baseline demographic, oncologic, and cardiovascular data were collected in a dedicated study database. The database was designed to integrate demographic, oncologic, and cardiovascular variables for each patient, allowing longitudinal correlation of data and comparative analysis across evaluation time points (T0–T3).

### Participant selection criteria

Patients were eligible if they met the following inclusion criteria:

- Age > 18 years;
- histopathologically confirmed oncologic diagnosis (included entities: breast cancer, Hodgkin and non-Hodgkin lymphomas, lung cancer);
- indication for chemotherapy or targeted therapy associated with a risk of cardiotoxicity;
- documented baseline cardiac function;
- absence of clinically manifest heart failure at baseline;
- signed informed consent.

Exclusion criteria:

- Pre-existing advanced heart failure (NYHA class III–IV);
- severe pre-existing arrhythmias;
- contraindications to radionuclide imaging;
- advanced renal disease (GFR < 30 mL/min/1.73 m²);
- severe comorbidities with major cardiovascular impact (e.g., acute myocardial infarction within the previous 3 months, untreated severe valvular disease) that could negatively affect the evaluation of cardiotoxicity.

### Evaluation protocols – measurement time points

Assessments were performed at the following time points:

T0 (baseline) – before initiation of antineoplastic therapy;

T1 (intermediate) – after 3–4 treatment cycles or approximately 6–8 weeks after therapy initiation (depending on the therapeutic protocol);

T2 – at completion of the planned antineoplastic therapy;

T3 (late follow-up) – approximately 6 months (up to a maximum of 12 months) after therapy completion to evaluate late effects.

At each time point, the following evaluations were performed: medical history, cardiovascular examination, ECG, and biomarker assessment.

Echocardiographic functional parameters were collected during follow-up when available and clinically indicated. These included left ventricular ejection fraction (LVEF) and global longitudinal strain (GLS), which were used to complement biomarker-based monitoring of subclinical cardiac dysfunction.

### Determination of molecular biomarkers

In the present study, three biomarkers were measured at T0, T1, T2, and T3: high-sensitivitycardiac troponin T (hs-cTnT), NT-proBNP, and soluble ST2 (sST2).

hs-cTnT was measured using the Elecsys^®^ Troponin T hs assay on the Roche cobas e platform, based on electrochemiluminescence immunoassay methodology, manufactured by Roche Diagnostics International Ltd. The upper reference limit corresponding to the 99th percentile was 14 ng/L.

NT-proBNP was measured using the Elecsys^®^ proBNP II assay on the Roche cobas e platform, based on electrochemiluminescence immunoassay methodology, manufactured by Roche Diagnostics International Ltd.

sST2 was measured using the Presage^®^ ST2 Assay, Critical Diagnostics, ELISA, FDA-cleared/CE-IVD, manufactured by Critical Diagnostics Inc. A threshold of 35 ng/mL was used for cardiovascular risk stratification.

Blood samples were collected under fasting conditions in standard biochemistry vacutainers, centrifuged at 3000 rpm for 10 min, and either analyzed immediately or stored at − 80 °C until processing.

The results were integrated into the main study database. Statistical analyses were performed using IBM SPSS Statistics v29.0 (IBM Corp., Armonk, NY, USA).

### Operational definitions

For uniform interpretation of the collected data, the terminology, parameters, and biomarkers used in the study were defined according to recent international guidelines (ESC Cardio-Oncology Guidelines 2022, ASE 2023, EANM) [7, 14, 21].

### Statistical methods

Statistical analysis was performed using IBM SPSS Statistics version 29.0 (IBM Corp., Armonk, NY, USA).

The distribution of continuous variables was evaluated using the Shapiro–Wilk test and visual inspection (histograms and Q–Q plots). Biomarkers showed an asymmetric distribution; therefore, non-parametric methods were used.

Continuous variables are expressed as median [IQR] or mean ± SD, depending on distribution, while categorical variables are presented as frequencies and percentages.

Correlation analyses were performed using Spearman’s rank correlation coefficient.

The temporal evolution of biomarkers (hs-cTnT, NT-proBNP, sST2) between time points T0–T3 was analyzed using the Friedman test for repeated measures. When a significant global result was obtained, pairwise comparisons were performed using the Wilcoxon signed-rank test for paired samples, with Bonferroni adjustment to control type I error.

In addition to longitudinal within-patient analyses, exploratory cross-sectional comparisons between oncologic subgroups were performed to evaluate potential differences related to treatment exposure.

Comparisons of biomarkers between the three oncologic subgroups at time points T2 and T3 were performed using the Kruskal–Wallis test. In cases of a significant overall result, pairwise comparisons were conducted using the Dunn test with Bonferroni correction.

The level of statistical significance was set at *p* < 0.05 (two-tailed).

### Ethical considerations and data security

The study was approved by the Ethics Committee of the “Prof. Dr. Alexandru Trestioreanu” Oncology Institute, Bucharest, in accordance with the Declaration of Helsinki (2013 revision) and European regulations regarding biomedical research involving human subjects.

All participants received detailed information regarding the purpose, procedures, potential benefits, and risks of the study and signed informed consent prior to enrollment.

Personal data were anonymized and coded using a unique identification system. Data storage was performed on secure servers.

Data processing and archiving complied with the requirements of Regulation (EU) 2016/679 on the protection of personal data (GDPR) and applicable national legislation. Data export and statistical analyses were conducted only on anonymized datasets.

All principles of biomedical research ethics were respected, and the standard therapeutic management of included patients was not modified in any way.

## Results

### Clinical and demographic characteristics of the study cohort

A total of 90 patients were consecutively enrolled in the study at the *“Prof. Dr. Alexandru Trestioreanu” Oncology Institute* in Bucharest, all of whom met the eligibility criteria defined in the research protocol.

The median age of the patients was 56 years (interquartile range [IQR] 48–64 years), and the majority were female (62%), consistent with the high proportion of breast cancer cases within the studied cohort. The distribution by sex and type of malignancy was as follows:

- Breast cancer – 30 patients (100% female);
- Lymphomas – 30 patients (53% female, 47% male);
- Lung cancer – 30 patients (33% female, 67% male).

During the follow-up period, six patients died from biological causes independent of the cardiovascular evaluation protocol.

The initial descriptive baseline analysis included all 90 patients.The longitudinal analysis of biomarker dynamics was performed on patients with complete data at all four evaluation time points (complete-case analysis, *n* = 84).

The six patients who did not complete follow-up were included in the baseline descriptive analysis but were excluded from the longitudinal analysis.

No data imputation methods were applied, given the relatively low proportion of missing data (6.7%). The potential impact of these exclusions is discussed in the Limitations section.

**Fig. 1:**
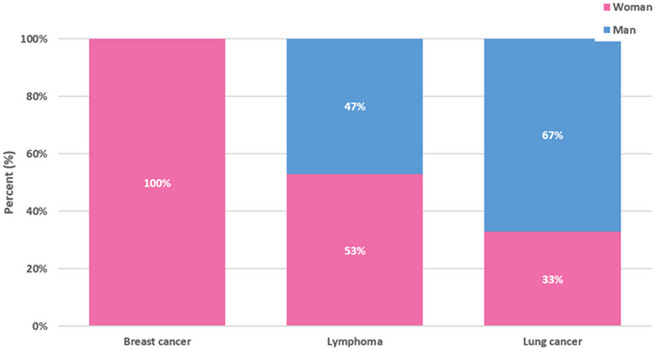
Sex distribution across oncologic subgroups

This distribution corresponds to the typical structure of oncologic pathology treated at the Bucharest Oncology Institute, where breast tumors and hematologic lymphomas predominantly occur in women, while lung neoplasms are more frequently observed in men (Fig. 1).

Baseline demographic, cardiovascular, oncologic, echocardiographic, radiotherapy, and biomarker characteristics are summarized in Table 1. Comparisons between the three oncologic subgroups were performed using the Kruskal–Wallis test for continuous non-normally distributed variables, one-way ANOVA for normally distributed continuous variables, chi-square test for categorical variables, and Fisher’s exact test when appropriate.

**Table 1:** Clinico-demographic characteristics of the study population (baseline T0)

Parameter	Breast cancer (*n* = 30)	Lymphoma (*n* = 30)	Lung cancer (*n* = 30)	Total (*N* = 90)	*p*-value	Statistical test
Age (years), median [IQR]	54 [46–62]	49 [38–60]	63 [56–69]	56 [48–64]	0,002	Kruskal–Wallis
Sex: Female, n (%)	30 (100%)	16 (53%)	10 (33%)	56 (62%)	< 0.001	Chi-square
Sex: Male, n (%)	0 (0%)	14 (47%)	20 (67%)	34 (38%)	< 0.001	Chi-square
BMI (kg/m²), mean ± SD	26,3 ± 3,9	25,7 ± 4,2	24,9 ± 3,6	25,6 ± 3,9	0.383	One-way ANOVA
ECOG 0–1, n (%)	27 (90%)	26 (87%)	24 (80%)	77 (86%)	0.533	Chi-square
Current smoker, n (%)	5 (17%)	7 (23%)	14 (47%)	26 (29%)	0.027	Chi-square
Arterial hypertension, n (%)	13 (43%)	10 (33%)	11 (37%)	34 (38%)	0.718	Chi-square
Dyslipidemia, n (%)	12 (40%)	9 (30%)	10 (33%)	31 (34%)	0.709	Chi-square
Diabetes mellitus, n (%)	5 (17%)	4 (13%)	5 (17%)	14 (16%)	0.919	Chi-square
Known coronary artery disease, n (%)	2 (7%)	2 (7%)	3 (10%)	7 (8%)	0.856	Fisher
Prior radiotherapy, any site, n (%)	18 (60%)	9 (30%)	3 (10%)	30 (33%)	< 0.001	Chi-square
Left-chest radiotherapy, n (%)	10 (33%)	0 (0%)	1 (3%)	11 (12%)	< 0.001	Fisher
Right-chest radiotherapy, n (%)	8 (27%	0 (0%)	1 (3%)	9 (10%)	0.001	Fisher
Mediastinal radiotherapy, n (%)	0 (0%)	9 (30%)	1 (3%)	10 (11%)	< 0.001	Fisher
Advanced stage (III–IV), n (%)	9 (30%)	18 (60%)	22 (73%)	49 (54%)	0.003	Chi-square
Baseline LVEF (%), mean ± SD	61 ± 5	60 ± 5	59 ± 6	60 ± 5	0.356	One-way ANOVA
Baseline GLS (%), mean ± SD	−19,8 ± 2,0	−19,6 ± 2,2	−19,0 ± 2,1	−19,5 ± 2,1	0.313	One-way ANOVA
Detectable hs-cTnT (> LoD), n (%)	3 (10%)	2 (7%)	4 (13%)	9 (10%)	0.690	Fisher
NT-proBNP (pg/mL), median [IQR]	105 [70–180]	120 [80–190]	145 [90–210]	125 [80–190]	0.210	Kruskal–Wallis
sST2 (ng/mL), median [IQR]	24 [19–31]	26 [20–34]	28 [22–37]	26 [20–34]	0.180	Kruskal–Wallis
Anthracyclines, n (%)	27 (90%)	18 (60%)	1 (3%)	46 (51%)	< 0.001	Chi-square
Cumulative doxorubicin dose (mg/m²), median [IQR]	240 [200–280]	220 [180–260]	–	230 [180–270]	0.120	Kruskal–Wallis
Anti-HER2 therapy (trastuzumab ± pertuzumab), n (%)	18 (60%)	0	0	18 (20%)	< 0.001	Fisher
Platinum/Taxanes, n (%)	8 (27%)	9 (30%)	22 (73%)	39 (43%)	< 0.001	Chi-square
TKI and/or ICI, n (%)	0	2 (7%)	20 (67%)	22 (24%)	< 0.001	Fisher exact

Values are expressed as mean ± standard deviation (SD) or median [interquartile range – IQR], or number and percentage, depending on variable type and distribution.

*“–”* not applicable, *BMI* body mass index, *ECOG* Eastern Cooperative Oncology Group performance status, *LVEF *left ventricular ejection fraction, *GLS* global longitudinal strain, *LoD* limit of detection, *hs-cTnT* high-sensitivity cardiac troponin T, *NT-proBNP* N-terminal pro-B-type natriuretic peptide, *sST2 *soluble suppression of tumorigenicity-2, *HER2 *human epidermal growth factor receptor 2, *TKI* tyrosine kinase inhibitor, *ICI* immune checkpoint inhibitor

The study population exhibited a balanced distribution across the three major subgroups, with an overall female predominance (62%), a median age of 56 years, and a significant prevalence of cardiovascular risk factors (hypertension 38%, dyslipidemia 34%, diabetes mellitus 16%) (Table 1).

Overall, cardiovascular and metabolic comorbidities were present in a substantial proportion of patients. The most frequent conditions included: arterial hypertension – 38%; dyslipidemia – 34%; type 2 diabetes mellitus – 16%; and active smoking – 29%.

Radiotherapy exposure was recorded according to treatment location. Prior radiotherapy at any site was documented in 30 patients (33%), including left-chest radiotherapy in 11 patients (12%), right-chest radiotherapy in 9 patients (10%), and mediastinal radiotherapy in 10 patients (11%).

From the perspective of administered oncologic therapy:

- Anthracyclines were used in 51% of patients, with mean cumulative doses equivalent to doxorubicin 240 mg/m²;
- Anti-HER2 therapy (trastuzumab ± pertuzumab) was administered in 27% of patients, exclusively in the breast cancer subgroup, either as sequential monotherapy or in combination with anthracyclines;
- Platinum- and taxane-based regimens were used in 43% of patients, predominantly in lung cancer and in certain lymphoma subtypes;
- Targeted therapy and immunotherapy (TKI/ICI) were administered in 24% of patients, particularly within the lung cancer subgroup.

Baseline imaging parameters showed preserved ventricular function at study inclusion, with a mean LVEF of 60 ± 5% and a mean GLS of − 19.5 ± 2.1%. Serum biomarker values at baseline are presented in Table 1.

### Longitudinal echocardiographic functional parameters

Longitudinal echocardiographic functional parameters were available during follow-up and are presented in Table 2. LVEF and GLS were assessed at baseline and during follow-up visits when available and clinically indicated.

**Table 2:** Longitudinal echocardiographic functional parameters

Parameter	T0 baseline	T1	T2	T3	*p*-value	Statistical test
LVEF, %, mean ± SD	60 ± 5	59 ± 5	58 ± 6	59 ± 6	0.048	Repeated-measures ANOVA
GLS, %, mean ± SD	−19.5 ± 2.1	−18.8 ± 2.3	−18.1 ± 2.5	−18.5 ± 2.4	0.012	Repeated-measures ANOVA
ΔGLS T0–T2, %, median [IQR]	*–*	*–*	1.4 [0.6–2.3]	*–*	*–*	*–*
Relative GLS reduction > 15%, n (%)	*–*	9 (11%)	17 (20%)	13 (15%)	0.041	Cochran’s Q test
LVEF reduction ≥ 10% points, n (%)	*–*	3 (4%)	6 (7%)	4 (5%)	0.368	Cochran’s Q test

Values are expressed as mean ± standard deviation (SD) or median [interquartile range – IQR], or number and percentage, depending on variable type and distribution

For ΔGLS and relative reductions, baseline T0 was used as reference; therefore, these variables were not applicable at T0. Less negative GLS values indicate worsening myocardial deformation

*“–”* not applicable, *LVEF* left ventricular ejection fraction, *GLS* global longitudinal strain, *SD* standard deviation, *IQR *interquartile range, *NA* not applicable

A progressive reduction in GLS was observed during treatment follow-up, with the lowest mean GLS value recorded at T2. Relative GLS reduction > 15% was observed in 9 patients (11%) at T1, 17 patients (20%) at T2, and 13 patients (15%) at T3.

### Analysis of serum biomarker dynamics in the entire patient cohort

The analysis of serum biomarker dynamics was performed in the cohort of 84 patients, monitoring the evolution of high-sensitivity cardiac troponin T (hs-cTnT), NT-proBNP, and soluble ST2 (sST2) at time points T0–T3 (Table 3).

These determinations were carried out synchronously with imaging assessments, allowing correlation analysis between biomarker changes and echocardiographic parameters.

**Table 3:** Evolution of the median values of cardiac biomarkers(*N* = 84)

Measurement moment	hs-cTnT (ng/L)	NT-proBNP (pg/mL)	sST2 (ng/mL)
T0 (baseline)	5 [3–8]	120 [80–190]	26 [20–34]
T1 (at 6–8 weeks.)	11 [7–19]	175 [120–270]	29 [23–37]
T2 (treatment end	15 [10–25]	210 [150–320]	33 [26–42]
T3 (6 months follow-up)	8 [5–13]	160 [110–230]	30 [24–39]

Values are expressed as median [interquartile range]

*hs-cTnT* high-sensitivity cardiac troponin T, *NT-proBNP* N-terminal pro-B-type natriuretic peptide, *sST2* soluble suppression of tumorigenicity-2

For the analysis of the temporal evolution of biomarkers (hs-cTnT, NT-proBNP, sST2) in the overall cohort and across oncologic subgroups, non-parametric tests for repeated measures were used, given the asymmetric distribution of the data.

The Friedman test was applied to compare biomarker values across the four time points (T0, T1, T2, T3), followed by post-hoc Wilcoxon signed-rank tests with Bonferroni correction to identify pairs of time points with statistically significant differences (Table 4).

**Table 4:** *–* Friedman and Wilcoxon test results for biomarker changes across T0–T3 (*N* = 84)

Biomarker	Friedman Test (T0–T3)	global *p*	Significant Post-hoc Comparisons (Wilcoxon, *p* < 0.05)	Key Observations
hs-cTnT	χ²(3) = 45,7	< 0,001	T0–T1 (*p* = 0,006); T1–T2 (*p* = 0,011); T2–T3 (*p* = 0,019)	Significant early increase, peak at T2; partial decrease at T3
NT-proBNP	χ²(3) = 38,3	< 0,001	T0–T2 (*p* = 0,002); T0–T3 (*p* = 0,021)	Gradual increase with partial persistence at T3.
sST2	χ²(3) = 14,8	0,015	T0–T2 (*p* = 0,037); T0–T3 (*p* = 0,041)	Delayed and persistent increase up to T3

*hs-cTnT* high-sensitivity cardiac troponin T, *NT-proBNP* N-terminal pro-B-type natriuretic peptide, *sST2 *oluble suppression of tumorigenicity-2

All three biomarkers demonstrated significant temporal variation (*global p* < 0.05).

The largest median change was observed for hs-cTnT, which showed an early increase at T1 and T2 (*p* < 0.001), followed by a partial decline at T3.

NT-proBNP exhibited a gradual increase, reaching peak levels at the end of therapy (T2). sST2 demonstrated a delayed pattern of evolution, with significant increases observed between T0–T2 and T0–T3.

**Fig. 2:**
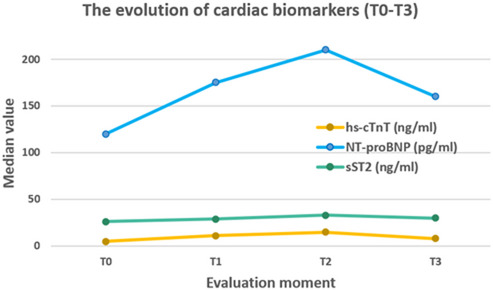
Evolution of the median values of cardiac biomarkers (hs-cTnT, NT-proBNP, sST2) between time points T0–T3. Values are expressed as medians [IQR]

The results of these statistical analyses are graphically illustrated in Fig. 2.

hs-cTnT increased from 5 [3–8] ng/L at baseline to 11 [7–19] ng/L at T1 and reached 15 [10–25] ng/L at T2, followed by a decrease to 8 [5–13] ng/L at T3.

NT-proBNP increased from 120 [80–190] pg/mL at T0 to 175 [120–270] pg/mL at T1 and 210 [150–320] pg/mL at T2, followed by lower values at T3.

sST2 increased from 26 [20–34] ng/mL at T0 to 29 [23–37] ng/mL at T1 and 33 [26–42] ng/mL at T2, with values of 30 [24–39] ng/mL at T3.

### Analysis by oncologic subgroups

The analysis by oncologic subgroups revealed relevant differences in biomarker values at T2 and T3.

To analyze differences between oncologic pathology subgroups at time point T2, the Kruskal–Wallis test was applied, followed by pairwise Dunn–Bonferroni comparisons (Table 5).

**Table 5:** Analysis of differences between oncologic subgroups at time point T2

Biomarker	χ²(2)	global *p*	Significant Differences (Dunn–Bonferroni, *p* < 0.05)
hs-cTnT	9,7	0,008	Breast cancer > Lymphomas (*p* = 0.031); Breast cancer > Lung cancer (*p* = 0.009).
NT-proBNP	5,3	0,071	Not significant; trend: Lymphomas > Lung cancer.
sST2	7,1	0,049	Lung cancer > Lymphomas (*p* = 0.042).

*hs-cTnT * high-sensitivity cardiac troponin T*, NT-proBNP *N-terminal pro-B-type natriuretic peptide*, sST2 *soluble suppression of tumorigenicity-2

**Fig. 3:**
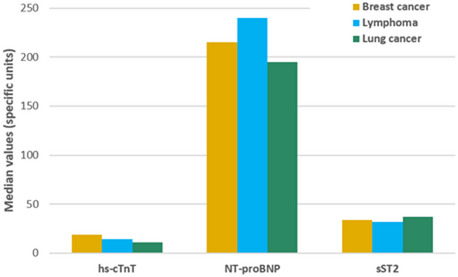
Comparison of median (T2) values of cardiac biomarkers across oncologic subgroups

For a more detailed comparative analysis, T2 values of the main biomarkers were evaluated across oncologic subgroups (Table 6; Fig. 3).

**Table 6:** *–* Median values of cardiac biomarkers at T2 across oncologic subgroups

Biomarker	Breast cancer (*n* = 29)	Lymphoma (*n* = 28)	Lung cancer (*n* = 27)	*p* (Kruskal-Wallis)
hs-cTnT (ng/L)	18,9 [13–28]	14,2 [9–21]	10,8 [7–17]	0,008
NT-proBNP (pg/mL)	215 [150–310]	240 [170–340]	195 [140–290]	0,071
sST2 (ng/mL)	34 [28–43]	32 [26–40]	37 [30–46]	0,049

Values are expressed as median [interquartile range]

*hs-cTnT* high-sensitivity cardiac troponin T, *NT-proBNP* N-terminal pro-B-type natriuretic peptide, *sST2* soluble suppression of tumorigenicity-2

At T2, hs-cTnT values were significantly different between subgroups (*p* = 0.008), with the highest median values observed in the breast cancer subgroup. NT-proBNP values did not differ significantly between subgroups (*p* = 0.071). sST2 values differed significantly between subgroups (*p* = 0.049), with the highest median values observed in the lung cancer subgroup.

At T3, biomarker values were also compared across oncologic subgroups using the Kruskal–Wallis test (Table 7).

**Table 7:** *–* Maximum median values (T3) of cardiac biomarkers across oncologic subgroups

Biomarker	Breast cancer (*n* = 29)	Lymphoma (*n* = 28)	Lung cancer (*n* = 27)	*p* (Kruskal–Wallis)
hs-cTnT (ng/L)	10,2 [6–15]	8,9 [5–13]	6,8 [4–10]	0,041
NT-proBNP (pg/mL)	165 [120–240]	185 [130–260]	150 [100–220]	0,067
sST2 (ng/mL)	31 [25–40]	32 [26–42]	35 [28–45]	0,052

Values are expressed as median [interquartile range]

*hs-cTnT* high-sensitivity cardiac troponin T, *NT-proBNP* N-terminal pro-B-type natriuretic peptide, *sST2* soluble suppression of tumorigenicity-2

### Correlation analysis

Correlation analysis was performed to evaluate associations between biomarker changes and echocardiographic functional changes.

A moderate inverse association was observed between changes in hs-cTnT and changes in global longitudinal strain (Δhs-cTnT vs. ΔGLS: *r* = − 0.42).

A moderate positive correlation was observed between NT-proBNP and sST2 levels (*r* = 0.48).

## Discussion

### Summary of key findings

The present prospective, observational, single-center cohort study evaluated the temporal dynamics of hs-cTnT, NT-proBNP, and sST2 in patients receiving potentially cardiotoxic antineoplastic therapy. The main findings were that hs-cTnT increased early during therapy, NT-proBNP showed a progressive increase during treatment, and sST2 displayed a later and more persistent elevation during follow-up. Differences in biomarker trajectories were observed across oncologic subgroups, and changes in hs-cTnT showed a moderate inverse association with changes in GLS.

Cardiotoxicity associated with antineoplastic therapy represents one of the major challenges of contemporary oncology, in the context of increasing survival rates and the diversification of therapeutic options [26].

The rationale for evaluating the dynamics of traditional cardiac biomarkers (troponin, NT-proBNP) and emerging biomarkers (ST2) at different stages of oncologic treatment was based on the hypothesis that elevated biomarker levels may anticipate the development of clinically manifest cardiac dysfunction in oncology patients.

### Study population and baseline cardiovascular risk

The study included adult patients with breast cancer, lymphomas, and lung cancer, malignancies commonly treated with regimens associated with a documented risk of cardiotoxicity (anthracyclines ± anti-HER2 therapy, platinum/taxane agents, tyrosine kinase inhibitors, and immunotherapy). The included patient group exhibited a balanced distribution across the three major subgroups, with an overall female predominance (62%), a median age of 56 years, and a significant prevalence of cardiovascular risk factors (hypertension 38%, dyslipidemia 34%, diabetes mellitus 16%).

The prevalence of cardiovascular comorbidities in our cohort was comparable to that reported in European cardio-oncology registries, where hypertension and dyslipidemia are frequently documented in patients undergoing cancer therapy [22–25].

The age structure and comorbidity profile are similar to those reported in international cardio-oncology studies such as SUCCOUR (echocardiography-guided cardioprotection) and ICOS-ONE (early troponin-guided prevention), which included patients with a median age of 55–60 years and a prevalence of hypertension between 35 and 45% [27, 28].

This similarity supports the clinical relevance of the cohort, although the single-center design limits generalizability.

### Biomarkers and multimodal monitoring in cardio-oncology

Until approximately a decade ago, cardiac monitoring relied almost exclusively on the late detection of a decline in left ventricular ejection fraction (LVEF). However, the development of molecular biomarkers has enabled the transition toward a proactive model of early detection and personalized prevention, capable of identifying myocardial injury in an early and potentially reversible phase.

Cardiac and molecular biomarkers are complementary to imaging methods, providing a biochemical perspective of the stages of myocardial injury. High-sensitivity cardiac troponin T (hs-cTnT) is an early indicator of cardiomyocyte injury. Early increases have been associated with a risk of cardiotoxicity up to eight times higher in several studies [29, 30].

NT-proBNP reflects hemodynamic stress and early diastolic dysfunction. Several studies, including the OVERCOME trial, demonstrated that increases in NT-proBNP during treatment preceded LVEF decline by 1–2 treatment cycles and were associated with the development of subclinical heart failure [10, 31, 32]. The ASE/EACVI recommendations also emphasize the value of natriuretic peptides in association with cardiac imaging parameters [33, 34].

In recent years, soluble ST2 (sST2), a member of the IL-33 receptor family, has been evaluated as a marker of inflammation and fibrotic remodeling. Clinical studies have shown that values exceeding 35 ng/mL are associated with persistent myocardial remodeling and unfavorable cardiovascular prognosis, particularly in heart failure populations [35–40].

### Temporal biomarker dynamics

The results obtained in the present study support the current pathophysiological model according to which antineoplastic therapy–induced cardiotoxicity represents a biological continuum involving cardiomyocyte injury, hemodynamic stress, and progressive fibrotic remodeling. Lyon and collaborators described this staged model, emphasizing that cardiac biomarkers reflect distinct phases of myocardial injury and should not be interpreted in isolation [41].

All three biomarkers demonstrated significant temporal variation during treatment and follow-up, supporting the presence of distinct biomarker trajectories. In the analyzed cohort, the early increase in hs-cTnT, followed by the rise in NT-proBNP and the late persistence of sST2 is broadly consistent with this biological sequence and supports the potential clinical value of serial biomarker monitoring.

High-sensitivity cardiac troponin T is currently considered the earliest biomarker of cardiomyocyte injury induced by oncologic therapies. Multiple longitudinal studies have demonstrated that troponin elevation during chemotherapy is associated with up to an eightfold increased risk of developing left ventricular dysfunction, depending on parameters such as the definition of cardiotoxicity (LVEF decline, CTRCD, clinical events), timing of troponin measurement, and type and intensity of treatment [29, 42–44].

In our study, hs-cTnT levels increased early during treatment, with peak values at T2. This pattern may reflect early myocardial injury during exposure to cardiotoxic oncologic therapies, particularly anthracycline-based and anti-HER2 regimens. At T3, hs-cTnT decreased compared with T2, although values remained higher than baseline in some patients.

Our results are consistent with previous studies, demonstrating significant hs-cTnT elevations during intermediate treatment phases, with peak values at the end of therapy. At T2, breast cancer patients treated with anthracyclines and anti-HER2 therapy exhibited the highest hs-cTnT values (median T2 values: breast cancer 18.9 ng/L [13–28 ng/L], lymphomas 14.2 ng/L [9–21 ng/L], lung cancer 10.8 ng/L [7–17 ng/L]), findings also reported in contemporary cardio-oncology studies [27, 43, 45–47].

Unlike troponin, NT-proBNP reflects ventricular wall stress and early ventricular dysfunction. Prospective studies have shown that progressive NT-proBNP changes may anticipate the development of heart failure even in the absence of LVEF changes [48–53]. An analysis published in Circulation demonstrated that the combined use of troponin and NT-proBNP significantly improves the predictive value for cardiotoxicity compared with each biomarker alone [49].

In our cohort, NT-proBNP increased gradually, reaching maximum values at the end of therapy (median values: T0 120 pg/mL, T1 175 pg/mL, T2 210 pg/mL, T3 160 pg/mL), suggesting progressive hemodynamic stress. This pattern may suggest increasing ventricular wall stress during treatment, although a causal mechanism cannot be established from the present data. The highest values were observed in lymphoma patients, may reflect a higher cumulative cardiotoxic burden due to anthracycline exposure and mediastinal radiotherapy. Similar findings have been reported in lymphoma survivor cohorts, demonstrating that anthracycline exposure, mediastinal radiotherapy, and cardiac radiation dose are associated with increased long-term cardiovascular risk, including heart failure, with combined exposure contributing to a greater cardiac burden [54–57].

A distinctive feature of this study is the inclusion of sST2, a biomarker of inflammation and fibrotic remodeling. Numerous studies have shown that sST2 levels ≥ 35 ng/mL are associated with activation of fibrotic and remodeling pathways and with an unfavorable cardiovascular prognosis, including increased mortality and hospitalization for heart failure [20, 58–61].

In the present study, sST2 demonstrated a delayed and more persistent pattern, increasing up to T2 and remaining elevated at T3. This delayed pattern may be compatible with persistent post-therapy inflammatory or remodeling processes.

Elevated sST2 levels were highest in lung cancer patients.This finding may be consistent with inflammatory effects associated with TKI and immune checkpoint inhibitors, therapies known to induce myocardial inflammation and structural remodeling even in the absence of marked acute injury [62–66].

However, sST2 interpretation in oncology patients requires caution because its values may be influenced by systemic inflammation, cancer type, age, comorbidities, and treatment exposure.

### Biomarker patterns across oncologic subgroups

In our cohort, patients treated with anthracyclines and anti-HER2 therapy, particularly those with breast cancer, exhibited a profile characterized by hs-cTnT elevation during treatment (T1–T2) followed by a moderate decrease after therapy completion. This observation is consistent with previous cardio-oncology studies showing that troponin elevation may occur during anthracycline- and anti-HER2-based therapy and may precede measurable decline in LVEF or GLS [27, 29, 42–47].

In lymphoma patients, NT-proBNP values were numerically higher at T2 and T3. This may reflect cumulative cardiotoxic exposure, including anthracycline-based regimens and prior mediastinal radiotherapy. Because the between-group differences for NT-proBNP did not reach statistical significance, this finding should be interpreted as exploratory. Similar findings have been reported in lymphoma survivor cohorts, demonstrating that both anthracycline exposure and cardiac radiation dose are associated with increased long-term risk of heart failure, with combined exposure contributing to a greater cardiac burden [54–57].

In lung cancer patients, sST2 values were higher, particularly at T2. Higher sST2 values in this subgroup may be associated with older age, cardiovascular comorbidities, systemic inflammation, and exposure to TKI and/or ICI therapy. Elevated sST2 levels in lung cancer patients may therefore suggest a remodeling- or inflammation-dominant profile, but this interpretation remains hypothesis-generating.

TKIs and immune checkpoint inhibitors have distinct cardiovascular toxicity profiles, including vascular toxicity, metabolic alterations, myocarditis, and immune-mediated myocardial inflammation [11–13, 62–66].

In cardio-oncology, the role of sST2 has recently been reinforced by studies demonstrating that combining sST2 with hs-cTnT and NT-proBNP significantly improves prediction of cardiotoxicity induced by anthracyclines and anti-HER2 therapies [18, 43, 67].

### Correlation between biomarkers and echocardiographic parameters

The addition of longitudinal echocardiographic parameters allowed assessment of the relationship between biomarker changes and myocardial functional changes. GLS showed a mild reduction during therapy, with the lowest mean value observed at T2.

Correlation analysis revealed a moderate inverse association between changes in hs-cTnT and changes in GLS, suggesting that increasing myocardial injury markers were associated with worsening myocardial deformation.

This association is consistent with recent studies demonstrating that GLS decline may precede LVEF reduction by 2–3 treatment cycles, occurring simultaneously with troponin elevation [43, 68–70]. Recent studies further demonstrated that early variations in troponin and myocardial strain act synergistically in predicting ventricular dysfunction, confirming that integrating inflammatory biomarkers and strain parameters can anticipate LVEF decline by approximately 6–8 weeks [43, 69, 71].

A moderate positive correlation was also observed between NT-proBNP and sST2. This may indicate a parallel increase of biomarkers associated with ventricular stress and remodeling. However, the strength of correlation was moderate, and these findings should be interpreted as associations rather than evidence of causality.

### Clinical implications

The statistical analysis performed in the present study supports the working hypothesis that each biomarker follows a distinct but interrelated kinetic pattern within the cardio-oncologic continuum: hs-cTnT as an early marker of myocardial injury, NT-proBNP as a marker of ventricular stress, and sST2 as a later marker associated with remodeling and inflammation.

This temporal sequence, demonstrated by Friedman tests and confirmed by post-hoc Wilcoxon analyses, supports the clinical utility of a multimodal approach to cardiac monitoring in oncology patients. However, the observed biomarker sequence should be interpreted as suggestive, not as definitive proof of a fixed biological cascade.

From a practical clinical perspective, hs-cTnT appears useful as an early warning signal (T1–T2) of myocardial injury, with good differentiation between subgroups at T2 (*p* = 0.008). NT-proBNP may provide information on cumulative ventricular load and stress, which may persist during follow-up even when acute injury decreases, while sST2 may add a dimension of remodeling and inflammation, increasing later and remaining more persistent.

The persistence of elevated sST2 levels at T3 supports the potential need for extended cardio-oncology monitoring beyond treatment completion. These findings support the integration of multimodal biomarker assessment and GLS analysis into routine surveillance strategies for early detection of oncotherapy-related cardiotoxicity in line with current ESC and ASE/EACVI recommendations [7, 14].

### Limitations and future perspectives

The limitations of this study include its single-center design and the relatively small sample size. Nevertheless, the prospective design and longitudinal evaluation enhance the robustness of the findings.

Six patients (6,7% of the cohort) did not complete the follow-up period due to death and were excluded from the longitudinal analysis, which was performed based on the principle of complete case analysis. The low proportion of missing data and the inclusion of all patients in the initial descriptive analysis maintain the coherence and consistency of the statistical estimates.

Another limitation is that echocardiographic follow-up parameters were collected when available and clinically indicated, which may introduce selection bias. The study also did not include cardiac magnetic resonance imaging, tissue characterization, or adjudicated clinical heart failure endpoints. Therefore, associations between biomarker trajectories and mechanisms such as myocardial injury, ventricular stress, inflammation, or remodeling remain suggestive rather than definitive.

The integration of biomarkers into predictive models based on machine learning, as proposed in recent literature, represents an important future research direction. Future studies should include larger multicenter cohorts, standardized imaging follow-up, longer observation periods, and clinically adjudicated cardiovascular outcomes.

## Conclusions

- High-sensitivity cardiac troponin T (hs-cTnT) showed an early increase during treatment and was moderately associated with changes in global longitudinal strain (GLS). These findings suggest that hs-cTnT, when interpreted together with echocardiographic parameters, may contribute to the early identification of subclinical cardiac changes during cancer therapy.
- NT-proBNP and sST2 showed distinct temporal patterns during treatment and follow-up. NT-proBNP increased progressively during therapy, whereas sST2 showed a later and more persistent elevation. These trajectories are suggestive of complementary cardiovascular responses to antineoplastic therapy, including ventricular stress and remodeling-related processes, but they do not confirm a definitive biological continuum.
- The combined assessment of hs-cTnT, NT-proBNP, and sST2 may provide more comprehensive information than any single biomarker alone, although predictive performance was not formally validated in the present study.
- The persistence of elevated sST2 levels six months after therapy may indicate ongoing remodeling-related or inflammatory processes, but its prognostic significance for residual cardiac dysfunction requires further validation.
- These findings support the potential value of a multimodal monitoring approach combining serial biomarkers with imaging parameters, particularly GLS, for the evaluation of subclinical cardiotoxicity in patients receiving potentially cardiotoxic cancer therapy.

## Data Availability

The datasets generated and/or analyzed during the current study are available from the corresonding authors on reasonable request.

## Abbreviations

BMI: Body Mass Index
ECG: Electrocardiogram
ECOG: Eastern Cooperative Oncology Group performance status
GLS: Global Longitudinal Strain
hs: cTnT–High–sensitivity cardiac troponin T
ICI: Immune Checkpoint Inhibitor
LVEF: Left Ventricular Ejection Fraction
NT: proBNP–N–terminal pro–B–type Natriuretic Peptide
sST2: Soluble Suppression of Tumorigenicity–2
TKI: Tyrosine Kinase Inhibitor
